# Revolutionizing dermatopathology using AI in skin diagnostics: scoping review

**DOI:** 10.3389/fmed.2026.1614681

**Published:** 2026-02-19

**Authors:** Rawan Rammal, Ahmad Mohy U Din, Tanvir Alam

**Affiliations:** College of Science and Engineering, Hamad Bin Khalifa University, Doha, Qatar

**Keywords:** AI, CNN, dermatology, LLM, ViT

## Abstract

AI models are becoming is increasingly used to enhance skin disease diagnosis and treatment. This scoping review complies with the PRISMA-ScR guidelines and after considering the inclusion and exclusion criteria, 12 articles published between 2017 and 2024 were considered. Majority of the publications are published from US and China. Among the selected studies, CNN- and ViT-based AI models were the most commonly used in literature, while LLM-based models (such as SkinGPT and Gemini-based models) appear in recently times more frequently to conduct interactive analysis for users. Recent studies have increasingly featured LLM-based models (e.g., SkinGPT, Gemini), indicating their growth as novel architectures in contrast to traditional CNN and ViT approaches. Among the diseases, the studied mainly covered melanoma, nevi, basal cell carcinoma, keratinocyte carcinoma, seborrheic keratosis, colorectal adenoma, etc. Our research reveals that while AI models excel in diagnosing prevalent and well-documented skin problems, their diagnostic efficacy significantly diminishes for rare or underrepresented diseases, highlighting the necessity for more robust, diversified, and clinically validated models. AI models are often too generic for multiple skin diseases. The studies utilized both private clinical data and public accessible resources, including ISIC and MoleMap. Majority of the AI models need improved clinical validation and regulatory standards covering ethical and legal standards to be considered as a tool for healthcare service providers. Despite these constraints, the reviewed studies indicate that AI models can enhance dermatopathology by increasing lesion classification precision, facilitating early detection, and reducing diagnostic strain; underscoring their prospective significance for clinicians and patients.

## 1 Introduction

Dermatopathology facilitates the distinction between inflammatory dermatoses, such as eczema and psoriasis, and neoplastic conditions, including melanoma and other skin malignancies, in the diagnosis of skin diseases (1). Dermatological diseases are widespread globally, with skin cancer being the most prevalent malignancy, and its frequency is on the rise, especially for non-melanoma skin cancers (2, 3). Skin diseases, especially skin cancer, are on the rise, while there are still not enough dermatologists to treat the population, especially in remote areas, which calls for new approaches that can help diagnose the diseases more effectively and promptly (4). AI models – such as Machine Learning (ML) and Deep Learning (DL) – is the most widely-discussed solution to overcome this challenge. Recent high-impact studies, such as (5), demonstrate the clinical utility of multimodal large language models in dermatology, combining visual and textual reasoning to improve diagnostic accuracy. The use of AI models in automatically analyzing medical images such as histopathological slides and dermoscopic images for skin conditions diagnosis has been investigated. Past studies have shown that AI models can be used in the identification of different skin related diseases. These models have been proven to provide high sensitivity in the identification of malignant tumors and differentiation of various skin diseases thus being useful for dermatologists (6, 39, 40). In the context of cutaneous melanoma, AI models must initially differentiate between melanocytic and non-melanocytic lesions. For melanocytic lesions, it is essential to evaluate critical histopathological parameters—silhouette and asymmetry, cellular distribution, mitotic count, and ulceration—to stratify risk and optimize pathologists’ workloads (7). Recent studies have highlighted the predictive significance of microRNA expression—specifically miR-21-5p and miR-146a-5p—as molecular markers that exhibit a substantial correlation with Breslow thickness in superficially spreading melanoma and may enhance standard histopathologic evaluation (8).

Nonetheless, various challenges hinder the integration of AI models in clinical practice, including inconsistent data quality, the absence of standardized performance metrics, and clinicians’ diminished confidence in AI-generated diagnostic recommendations—such as lesion classification, malignancy risk evaluation, and triage recommendations (9–11). There has never been a greater need for efficient and accurate AI models in dermatopathology as the field of AI models progresses and provides new ways to enhance diagnostic results, diminish the burden on clinicians, and provide better access to dermatological services (12). The main research question for this scoping review is: This paper aims at identifying the strengths, weaknesses and the opportunities and challenges of applying AI models in dermatopathology in across various clinical and research settings. This question form is very general which allows to identify the current state of research and what topics are missing (13). This review is intended to give an overview of the current state of AI models in dermatopathology focusing on the potential of current AI models in skin disease diagnosis based on dermatopathology images. It will discuss the problems that arise when trying to implement AI models into the clinical settings and outline potential avenues for research that may help overcome these problems (12).

### 1.1 Motivation

The reasons for investigating the application of AI models in dermatopathology are driven by the rising incidence of skin diseases worldwide, and therefore, the pressing demand for practical, accurate, and timely diagnostic approaches (14). Skin diseases, including chronic inflammatory conditions like eczema and psoriasis, are widespread globally and can impose a significant disease burden, especially in their moderate-to-severe symptoms (15). Due to the current scarcity of dermatology professionals including pathologists across the world especially in rural and hard-to-reach areas, there is a need to come up with new technologies that can assist dermatologists and pathologists in coming up with better solutions in diagnosing skin diseases. There are already some of these pressures which can be eased. Thanks to AI models in the form of automated tools that can help in the identification and classification of different types of skin diseases at an early stage (16). Dermatology is a field that has seen an upturn in medical imaging datasets which include dermoscopy images to support AI models in dermatopathology. If the same is employed for training AI models, then these datasets can enable the formation of the basis of diagnostic systems that may be as good or even better than human diagnosis in certain contexts (17). For example, CNNs have been employed for histopathological slides and dermoscopic images for identifying several skin lesions such as melanoma, basal cell carcinoma, psoriasis, and other inflammatory skin diseases. In this way, using AI-based models can help doctors find diseases faster and more correctly, which can improve the care for patients and make healthcare systems less busy (18, 19).

This review aims to assess the current state of AI models use in dermatopathology and how the existing problems faced by dermatologists and pathologists in the diagnosis of skin diseases may be solved by these systems. This review also seeks to determine the actual application of the AI models in real-world clinical practice and the possibility of AI models to enhance the skills of human clinicians to enhance better results for patients with dermatological illnesses.

### 1.2 Literature review

Artificial intelligence has transformed diagnostic approaches in dermatology and dermatopathology by aiding accurate identification of various skin conditions (20). Some of the areas in which AI models has been seen to provide accurate diagnosis include the more ordinary diseases such as eczema and psoriasis and the severe ones such as melanoma. This can help doctors cut off some of their work and make care more accessible for patients, especially in places where there aren’t many dermatologists. The increasing demand for dermatological services and scarcity of dermatologists along with the use of AI models in identifying and categorizing skin conditions has become important (21, 22). To contextualize the presented findings, it is essential to differentiate between the various AI models employed in dermatology and dermatopathology. Convolutional Neural Networks (CNNs) and Deep Neural Networks (DNNs) are image-centric models developed for classification tasks utilizing dermoscopic or histopathology images (23). Conversely, Large Language Models (LLMs) and emerging Large Multimodal Models (LMMs)—including GPT-4, SkinGPT-4, and Gemini—integrate image inputs with natural-language reasoning to produce diagnostic recommendations, clinical descriptions, or triage outputs (24). This methodological distinction is relevant as CNN/DNN models are designed for pixel-level pattern recognition, while LLM/LMM systems mimic facets of clinical reasoning by synthesizing visual and textual information (25). Classifying the literature by these model families clarify the advantages and disadvantages of conventional image-centric models in comparison to contemporary multimodal techniques.

Laohawetwanit et al. (26) used GPT-4 to detect colorectal adenomas in histopathological images. GPT-4 had some potential with sensitivity of 74% and specificity of 36%. Even though it was able to detect some of the lesions in the current study, its sensitivity and specificity should be improved to make it more suitable for wider application. The study also revealed that the model is dependent on the expert’s advice, which is a drawback in fully automated clinical scenarios. In the same manner, Hart et al. (22) evaluated CNN to categorize melanocytic lesions. The present study noted that when the CNNs were trained on carefully selected samples, they were able to achieve 99% in terms of accuracy rates; however, the accuracy rate reduced to 92% when the test was conducted on WSIs, with a sensitivity of 85% and a specificity of 99%. This performance drop shows the challenges that CNNs have when dealing with larger and more complicated data sets and underlines the importance of the model to control the data variety. In their cross-sectional study, Polesie et al. (27) asked dermatopathologists from around the world to share their thoughts on the use of AI models.

Although, 81.5% of the participants said that AI models was useful, only 18.8% of the participants said that they had a good or excellent knowledge of AI. This research gap points to the importance of enhancing the existing educational courses to enhance dermatologists’ awareness of AI models. Also, 42.6% of the participants thought that AI could assist in diagnosing skin tumors, while 62.1% acknowledged that AI models could be useful for automated mitosis detection to support its applicability in dermatology. Olsen et al. (28) discussed the use of CNNs in dermatopathological assessments including conventional and Spitz nevi. They discovered that CNNs obtained 99.0% accuracy on carefully selected image patches while this was only 52.3% on non-selected ones. On WSI the performance was better as CNNs had an accuracy of 92%, sensitivity of 85% and specificity of 99%. The study showed that, in 94% of the cases, the Spitz lesions were misdiagnosed as conventional melanocytic lesions thus highlighting the importance of large high quality and diverse datasets for training CNNs.

Hekler et al. (29) aimed to determine the ability of DNNs in the classification of dermoscopy images of different skin lesions. The DNN gave excellent results with 100% sensitivity and 98.9% specificity for nodular basal cell carcinoma. It also yielded a high sensitivity of 98.8% and a specificity of 100% in dermal nevi and sensitivity and specificity of 100% in seborrheic keratosis. These results show that DNNs have the promise to enhance the diagnostic sensitivity and specificity in dermatopathology. Esteva et al. (21) explained how CNN can accurately identify different forms of skin lesions. The model was trained on a dataset of 129,450 clinical images of over 2,000 types of skin disease. The CNN achieved a sensitivity of 91.0% and specificity of 88.9% in detecting malignant lesions which indicates that CNNs can provide diagnostic performance comparable to dermatologists. This work focuses on the possibility of utilizing deep learning models in clinical environments on a massive scale. Specifically, (30) conducted a multicentric study to test the ability of deep-learning models to predict whether slides contain histology from normal, inflammatory, neoplastic or other skin conditions. They used a ResNet50-based model, the model produced F1 score of 0.93, sensitivity of 0.92; specificity of 0.97; and the AUC was 0.99 for the detection of melanoma. Additionally, the work also created heat maps to explain the important regions in the images thus increasing the confidence of physicians in the AI models for clinical decisions.

Zhou et al. (31) presented SkinGPT-4, a large language model for dermatology diagnosis. Clinical severity, amateur photos, and unclean derma were excluded in the study, and the remaining 150 real-life cases were diagnosed by certified dermatologists; 78.76% of the diagnoses made by SkinGPT-4 were accurate or relevant. The system also had 83.13% for usefulness and 85% for helping the doctors in diagnosis. These results provide evidence of the feasibility of using LLMs to provide immediate and engaging assistance in clinical contexts. Panagoulias et al. (32) created Dermacen Analytica, an improved AI model based on machine learning that can help teledermatology doctors do better diagnoses. The system achieved a mean score of 0.87 for context and diagnostic information. Performance for multimodal or report-generation models is frequently assessed using ROUGE measures, namely ROUGE-1, ROUGE-2, and ROUGE-L, which quantify the overlap between the generated text and actual clinical reports (25). Elevated ROUGE scores signify superior correspondence with expert-written descriptions, enabling these measures appropriate for evaluating LLM-based dermatology systems (33).

The MIMIC-CXR dataset had R-1 of 0.2814, R-2 of 0.1334, and R-L of 0.2259, which are impressive results of the multi-modal AI approach in dermatology, especially for teledermatology.

Zhang et al. (34) improved Dermacen Analytica by integrating large language models with machine learning methods. Gemini models that the authors used in their work produced R-1 scores of 0.2814, R-2 scores of 0.1334, and R-L scores of 0.2259 on the MIMIC-CXR dataset. These models performed very well in identifying the lesions and in producing reports and underscore the potential of incorporating AI model for better dermatological outcomes. To tackle the OOD images and provide hierarchical classification in dermatopathology, Mehta et al. (35) introduced the Hierarchical-Out Distribution-Clinical Triage (HOT) model. According to this model, F1 scores increased from 0.574 to 0.607 by using hierarchical predictions in the model. The F1-score was raised to 0.617 when the clinical and the dermoscopic image datasets were combined indicating its utility in real-life scenarios. The model also could handle OOD data and obtain the AUROC of 81.80% for OOD(17 cL) and 71.79% for OOD.

Naeem & Anees (36) presented DVFNet, a deep learning model for the classification of skin conditions using dermoscopy images. Traditional deep learning models like VGG-16, AlexNet, and ResNet-50 were not as accurate as this model, which got a score of 98.32% on the ISIC 2019 dataset. It also had a high precision of 98.23%, recall of 98.19%, and F1 score of 98.23% proving the efficiency of the model in recognizing various skin conditions.

### 1.3 Gap in literature

Despite substantial progress in AI for dermatopathology, certain limitations persist. Although multiple studies indicate that AI models may detect various skin disorders, the area still lacks thorough evaluations that measure performance across both neoplastic and inflammatory states using standardized criteria. Esteva et al. (21) demonstrated CNN-based models may effectively identify prevalent skin cancers; however, their efficacy significantly diminishes if faced with rare situations or images of inconsistent quality. These findings emphasize the necessity for extensive validation of AI models across varied, real-world healthcare environments.

Hart et al. (22) Similarly, it was noted that CNNs exhibit strong performance on curated datasets but experience significant declines when applied to whole-slide images or larger, more heterogeneous datasets, highlighting ongoing challenges with model transferability and the need for standardized benchmarking in dermatopathology. Initial multimodal and LLM-based systems encounter similar constraints: Laohawetwanit et al. (26) discovered that GPT-4 attained merely moderate sensitivity (74%) and low specificity (36%), with performance significantly reliant on expert prompting, thereby restricting its applicability for complete automation, especially in resource-limited clinical environments.

Clinical readiness is also hindered by inadequate clinician knowledge and confidence. In a worldwide survey, Polesie et al. (27) indicated that while the majority of dermatopathologists regarded AI as beneficial, only 18.8% said they possessed a good or excellent comprehension of these techniques, revealing a significant educational deficiency. Data quality is an ongoing challenge; for instance, Olsen et al. (28) exhibited robust CNN performance on curated image patches, although significant accuracy declines were observed using non-curated inputs, emphasizing the necessity for diverse, high-quality datasets in both training and validation phases.

Hekler et al. (29) observed that while deep neural networks (DNNs) have shown good accuracy in detecting basal cell carcinoma and other lesions, their efficacy on novel or untested cases is inadequately confirmed, highlighting the necessity for larger and more multifaceted cohorts (30) expressed similar concerns, exhibiting robust melanoma detection capabilities with a ResNet50-based model while emphasizing that restricted interpretability hinders physician adoption—a persistent problem in numerous deep-learning systems.

Emerging LLM and multimodal models encounter similar issues. Zhou et al. (31) reported that SkinGPT-4 achieved an accuracy of 78.76% on real-world instances, although it encountered challenges with unusual diseases and required further refinement prior to clinical application. Similarly, Panagoulias et al. (32) showed favorable efficacy using Dermacen Analytica in teledermatology; however, the study did not assess its applicability for rare diseases, resource-limited contexts, or multi-center clinical settings. The lack of strong external validation further restricts the generalizability of these systems.

Zhang et al. (34) combined Dermacen Analytica with LLMs to enhance diagnostic accuracy; however, the study did not investigate the performance of these hybrid models in actual clinical processes, including issues pertaining to data variability and physician acceptance. Mehta et al. (35) introduced the HOT model to tackle out-of-distribution data; nonetheless, issues regarding its interpretability and efficacy across diverse datasets persist, hindering its suitability for widespread clinical application. Naeem and Anees (36) proposed DVFNet for dermoscopic image classification; nonetheless, the model necessitates validation on bigger, more heterogeneous datasets, especially those with underrepresented or noisy clinical cases. Notably, none of these models have undergone thorough multi-center or prospective clinical validation, leaving their real-world reliability largely unverified. These investigations highlight persistent shortcomings in dataset diversity, model interpretability, real-world validation, and physician acceptance. Resolving these difficulties is crucial for developing AI systems that are resilient, generalizable, and applicable to standard dermatopathology practice.

### 1.4 Contribution of work

This review adds to the current literature on AI models in dermatopathology by summarizing the current literature and identifying gaps that require further investigation. Specifically, this work:

- *Summarizes current applications*: It presents data on the application of AI models in dermatopathology to diagnose such conditions as lesion detection, classification, and disease identification. This is a useful review for those involved in education, practice, and research of dermatology to consider the use of AI models and the potential impact it may have. The role of AI models in increasing diagnostic precision is highlighted, with examples of applications in different skin diseases including malignant as well as benign neoplasms. More than that, the review shows how AI models can be used to make routine chores easier, like analyzing medical images and pathology slides, which can help dermatopathologists make faster and more accurate diagnoses. In this way, AI models could also help avoid misdiagnosis and increase the efficiency of the service in the regions where dermatological facilities are scarce.
- *Identifies gaps in research*: This review also discusses some limitations in current literature, including the lack of a unified approach to the evaluation, the lack of diverse and comprehensive data sets, and the lack of clarity around the decision-making process in AI models. This analysis will assist other future studies to address the challenges of applying AI models in clinical practices. A major challenge is developing AI models that would apply to a range of patient populations and therefore effective for different patient populations. The review also focuses on the issue of explainability which is critical as clinicians are expected to rely fully on the predictions made by these AI models. If these issues are not addressed, AI models may continue to be perceived as a device that clinicians are unable to effectively use in their practice owing to concerns about the tool’s credibility and openness.
- *Proposes future directions*: The review proposes several possible directions for the development of AI models in dermatopathology. This paper draws attention to the fact that the integration of AI models with other forms of data can enhance diagnostic accuracy. The review also emphasized the fact that there are no standard performance benchmarks for the AI models, thus hampering the comparison of studies and clinical applications. This standardization will ensure more frequent utilization of AI tools as their efficacy will be validated. In addition, the review promotes designing AI models that can work in different contexts, including the best-equipped hospitals to the scarce-resource settings, hence, very useful in applying AI in healthcare.
- *Serves as a framework for implementation*: This study describes how to straightforwardly understand the opportunities and challenges of utilizing AI models in dermatopathology. In light of recent research and based on maintaining the criteria for evaluations, this review provides doctors and educators with guidelines on how best and safely apply AI technologies. This also fosters the need for more work with other institutions to develop AI models that can be integrated into various healthcare systems so that the AI tools can be implemented in various healthcare facilities.

Overall, this review synthesizes recent applications of AI models in dermatopathology, identifying key performance outcomes, limitations, and areas for future research. The study suggests ideas that may revolutionize the diagnosis of skin conditions worldwide by highlighting current shortcomings and offering solutions. These results might help policymakers make the best rules for AI models use in healthcare, making sure that progress is made in an honest and fair way that does not leave any communities behind.

## 2 Methods

### 2.1 Materials and methods

This scoping review aimed at identifying the current state of using artificial intelligence (AI) in dermatopathology for the diagnosis of skin diseases. This research adhered to the PRISMA-ScR (Preferred Reporting Items for Systematic Reviews and Meta-Analyses Extension for Scoping Reviews) principles to guarantee methodological transparency and replicability. The evaluation method encompassed formulating the research question, identifying relevant research, selecting eligible studies based on established PICO criteria, charting the data, and summarizing the findings.

**Figure 1:**
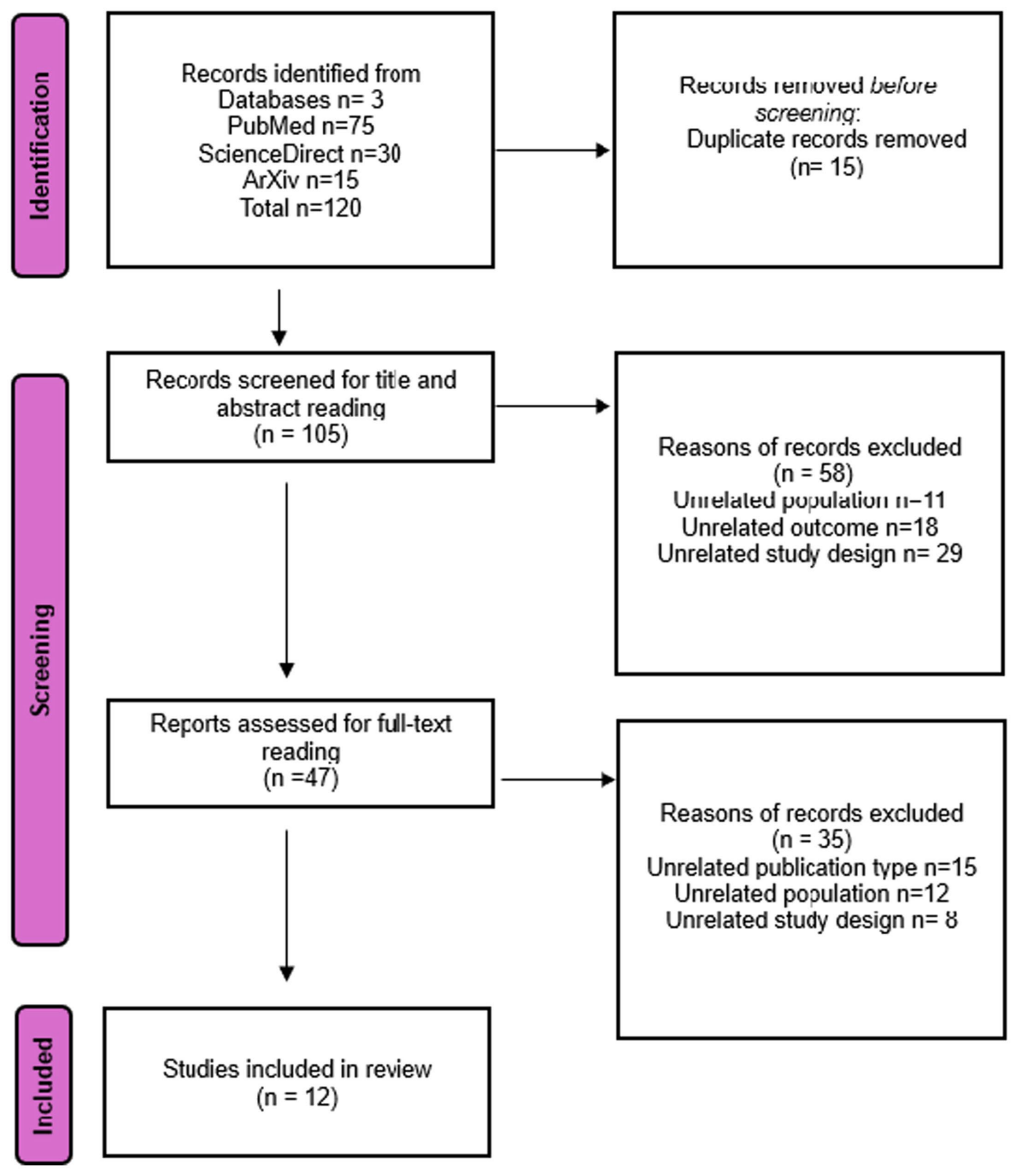
Study selection process using PRISMA guideline.

The overall flow of the study is highlighted in Figure 1.

### 2.2 Search strategy

#### 2.2.1 Search sources

This review explored three primary information sources for relevant literature: PubMed, ScienceDirect, and the ArXiv repository. These databases were chosen for their comprehensive indexing of biomedical literature, clinical dermatology research, pathology research, and innovative artificial intelligence papers important to dermatopathology. The search was performed in November 2024, with the date range confined to papers published from 2017 to 2024 to encompass recent breakthroughs in AI within dermatopathology.

In addition to the primary database searches, the reference lists of all included articles were manually reviewed to identify additional studies that met the eligibility criteria but were not initially identified in the search results. This approach facilitated a more thorough gathering of relevant research, especially concerning emerging AI technologies that might be published in preprints or interdisciplinary publications.

#### 2.2.2 Search terms

We established the search terms by examining the current literature on artificial intelligence in dermatology and pathology, ensuring alignment with prevalent terminology in the discipline. The search phrases were chosen based on three fundamental concepts: (1) the intervention, encompassing AI methodologies including machine learning, deep learning, convolutional neural networks, and large language models; (2) the target domain, concentrating on dermatopathology, skin diseases, and associated diagnostic tasks; and (3) the outcomes, highlighting diagnostic performance metrics such as accuracy, sensitivity, specificity, and classification efficacy. The complete search terms and strings used for each database and category are presented in Table 1.

**Table 1:** Search terms/strings for each category and database.

Category	Type	Search terms/Strings
	Technology	“Artificial intelligence,” “Machine learning,” “Deep learning,” “Large Language Models”
	Disease	“Skin cancer,” “Melanoma,” “psoriasis,” “Dermatopathology”
	Outcome	“Diagnostic accuracy,” “Sensitivity,” “Specificity,” “Classification”

Database	Database name	Database query	Searched results
	PubMed	((“Artificial intelligence”[Title/Abstract] OR “AI”[Title/Abstract]) AND (“Machine learning”[Title/Abstract] OR “Deep learning”[Title/Abstract]) AND (“Dermatopathology”[Title/Abstract] OR “Skin diagnostics”[Title/Abstract]) AND (“Diagnostic accuracy”[Title/Abstract] OR “Efficiency”[Title/Abstract]) AND (“2017/01/01”[Date - Publication]: “2024/12/31”[Date - Publication]) AND (“English”[Language]))	75
	Science Direct	(“Artificial intelligence” OR “AI”) AND (“Machine learning” OR “Deep learning”) AND (“Dermatopathology” OR “Skin diagnostics”) AND (“Diagnostic accuracy” OR “Efficiency”)	30
	ArXiv	(“Artificial intelligence” OR “AI”) AND (“Machine learning” OR “Deep learning”) AND (“Dermatopathology” OR “Skin diagnostics”) AND (“Diagnostic accuracy” OR “Efficiency”)	15

### 2.3 Eligibility criteria

The inclusion criteria for the review were as follows:

1. *Population*: Studies involving human dermatological conditions assessed through dermatopathology, including both inflammatory skin diseases and skin cancers.
2. *Intervention*: Studies applying artificial intelligence models, such as machine learning, deep learning, convolutional neural networks, large language models, or multimodal AI, for diagnostic classification, lesion identification, or decision-support in dermatopathology.
3. *Comparator*: Not required; studies may compare AI performance to clinicians, other AI models, or no comparator.
4. *Outcomes*: Studies reporting diagnostic performance metrics (e.g., accuracy, sensitivity, specificity, AUC, F1 score) or demonstrating the impact of AI on diagnostic decision-making or workflow support.
5. *Study design*: Primary empirical studies using quantitative or mixed-methods designs.
6. *Language and time frame*: English-language articles published between 2017 and 2024.

Exclusion criteria:

1. *Population*: Studies that assess AI models on populations unrelated to skin diseases were excluded.
2. *Intervention*: Studies lacking of an AI component or those utilizing solely synthetic or simulated data were eliminated.
3. *Comparator*: Studies that provided no diagnostic assessment or reference standard were excluded.
4. *Outcomes*: Studies lacking diagnostic performance indicators such as accuracy, sensitivity, specificity, or any measure of clinical impact.
5. *Study design*: Studies that are not primary empirical research, including systematic reviews, meta-analyses, theoretical papers, editorials, concept papers, and randomized controlled trials.
6. *Language and time frame*: Studies not written in English or published outside the 2017–2024 timeframe.

### 2.4 Study selection

Two reviewers (RR) and (AM) individually reviewed all records obtained from the database searches. In the initial phase, titles and abstracts were assessed to determine preliminary relevance according to the established PICO eligibility criteria. Articles considered suitable or questionable at this stage were progressed to full-text screening. Discrepancies between the two reviewers regarding inclusion or exclusion were resolved through discussion until a consensus was achieved. Inter-rater reliability was evaluated using Cohen’s kappa (*κ* = 0.53), revealing moderate agreement.

A study by Laohawetwanit et al. (26) evaluated GPT-4 for the diagnosis of colorectal adenomas, rather than skin conditions. This study was selected due to its unique and pertinent evidence on GPT-4’s performance in histopathological image interpretation, a modality also utilized in dermatopathology. The article is among the initial assessments of a multimodal LLM applied to actual microscopic tissue images, employing tasks comparable to those in dermatopathology; specifically, lesion detection, morphological pattern recognition, and subtype categorization. The findings on diagnostic inconsistency (κ = 0.06–0.11), reliance on expert prompting, and constraints in image-based reasoning directly reveal the difficulties associated with implementing matching LLM systems for skin biopsies and dermatopathology procedures. The article offers methodological insights that are conceptually relevant to the objectives of this review, as studies on dermatopathology-specific LLMs are limited.

### 2.5 Selection of sources of evidence

Rayyan software filters the articles obtained from the databases and the repository. The selection process followed the PRISMA flow diagram, which included four steps:

1. *Identification*: 120 articles were gathered from searches in PubMed, ScienceDirect, and the ArXiv repository.
2. *Screening*: After eliminating 15 duplicate articles, 105 were left for title and abstract screening. Two reviewers independently evaluated the articles for relevance, discarding 58 articles that did not fit the inclusion criteria.
3. *Eligibility*: A full-text review of the 47 articles that passed the initial screening was performed. Eight articles were excluded due to lack of relevance, incomplete information, or insufficient methodological details.
4. *Inclusion*: Ultimately, 12 articles were selected for analysis after fulfilling all inclusion criteria.

### 2.6 Data extraction

Data was abstracted from the medical records using a pre formatted Excel data extraction form for the sake of standardization and completeness of the information collected. The subsequent categories were employed for the data extraction process:

#### 2.6.1 Publication information

- Author(s)
- Year of publication
- Source (journal name or repository)

#### 2.6.2 Study characteristics

- Country of the study
- Study design (e.g., quantitative, qualitative, mixed-methods)
- Disease type (e.g., melanoma, psoriasis, skin cancer, other dermatological conditions)
- Target population (e.g., age, gender, demographics)

#### 2.6.3 AI features

- Type of AI model used (e.g., CNNs, DNNs, LLMs, GPT-4, ResNet50)
- Dataset source (e.g., curated datasets, public datasets, clinical datasets)
- Diagnostic methodology (e.g., histopathological image analysis, dermoscopic image classification)
- AI models evaluation methods (e.g., sensitivity, specificity, accuracy)

#### 2.6.4 Outcomes

- Performance metrics: sensitivity, specificity, accuracy, Area Under the Curve (AUC), and F1 score
- Impact on clinical workflows (e.g., improved diagnostic speed, decision support)

#### 2.6.5 Barriers and challenges

- Data quality issue (e.g., noisy or unstructured data)
- Model limitations (e.g., interpretability, generalizability)
- Clinician trust and acceptance of AI models
- Diversity of datasets (e.g., demographic representation, underrepresented conditions)
- Ethical or regulatory challenges

### 2.7 Synthesis of results

The findings from the selected papers were grouped into different themes to understand the information presented. The first group was based on the AI models applications, categorizing the studies according to their primary objectives, which included lesion characterization, decision-making assistance, and patient involvement in the treatment. The second group discussed the characteristics of the AI models, the various categories, including CNNs, Transformers, and LLMs, the training techniques utilized, as well as the datasets used. Sensitivity, specificity, accuracy, and AUC were assessed to compare how well the models did across various studies. The main challenge and barrier were found to be related to problems such as bias in the dataset and limited generalizability, in addition to ethical concerns that hinder the advancement of AI models in dermatopathology. Lastly, the clinical impact was discussed to demonstrate how AI models can increase the speed of diagnosis, positively affect the workflow, and improve patient outcomes. These organized categories became the framework for the presentation of the results in the Results section of the paper (Table 2).

**Table 2:** Data extraction sheet.

Category	Extracted variables
Publication information	Author(s) Year of publication Journal or source name
Study characteristics	Country of the study Study design Disease type Target population
AI features	Type of AI model used Dataset source Diagnostic methodology AI evaluation methods
Outcomes	Performance metrics Impact on clinical workflows
Barriers and challenges	Data quality issue Model limitations Clinician trust and acceptance of AI models Diversity of datasets Ethical or regulatory challenges

## 3 Results

Twelve articles were included in this scoping review and focused on the application of AI models in dermatopathology, its issues, and impact on practice. The findings are presented under different categories including: AI models applications, AI model features, performance metrics, barriers and challenges, and impact on clinics.

### 3.1 AI applications

AI applications were divided into three primary groups: diagnostic tasks, decision support, patient engagement, and report generation. A large number of papers focused on diagnostic tasks such as lesion classification and detection while only a few papers discussed decision-making systems and patient interaction. A summary of the specific AI applications identified in the 12 studies is presented in Table 3.

**Table 3:** Overview of AI uses in different research.

Study	Reference	AI application	Type of task	Specific focus
1	Laohawetwanit et al. (26)	Diagnostic tasks	Image classification	Colorectal adenoma detection
2	Hart et al. (22)	Diagnostic tasks	Whole-slide analysis	Classification of melanocytic lesions
3	Polesie et al. (27)	Decision support	Analysis of survey based AI models potential in dermatopathology tasks	AI models attitudes toward certain tasks were defined, which include automated tumor margin detection, mitosis detection, and immunostaining evaluation.
4	Olsen et al. (28)	Diagnostic tasks	Binary classification	Basal cell carcinoma, dermal nevi, seborrheic keratosis
5	Hekler et al. (29)	Diagnostic tasks	Multimodal analysis	histopathological melanoma images into melanoma and nevus
6	Esteva et al. (21)	Diagnostic tasks	Multiclass classification	Skin lesion categorization: keratinocyte carcinoma, melanoma and other malignant and benign conditions.
7	Xie et al. (30)	Diagnostic tasks	Multiclass classification	melanoma and various nevi classification using whole-slide images and CNN visualization
8	Zhou et al. (31)	Patient engagement	Interactive diagnostic support	Multimodal analysis with text/image integration and treatment suggestions
9	Panagoulias et al. (32)	Diagnostic tasks, Decision support	Multimodal analysis	large language models and vision transformers integration for nuanced skin lesion diagnostics and tele-dermatology
10	Zhang et al. (34)	Diagnostic tasks, report generation	Multimodal analysis	medical imaging and text reports integration by GPT and Gemini-series models classify diseases and lesion segmentation
11	Mehta et al. (35)	Decision support, Diagnostic tasks	Hierarchical classification and triage recommendations	Classification of multi-level skin lesion using clinical and dermoscopic images, along with out-of-distribution (OOD) detection and clinical triage suggestions, to improve diagnostic accuracy.
12	Naeem & Anees (36)	Diagnostic tasks	Multiclass lesion classification	Skin cancer classification in the initial stage in eight categories

### 3.2 AI model features

The studies employed various types of AI modeling techniques including Convolutional Neural Networks (CNNs), and Large Language Models (LLMs). Table 4 offers a thorough comparison of the model architectures, dataset sources, and diagnostic methodologies.

**Table 4:** Comparison of model characteristics across studies.

Study	Reference	AI model	Dataset source	Diagnostic methodology
1	Laohawetwanit et al. (26)	GPT-4	100 polyp images (50 adenomas, 50 non-adenomas)	Histomorphology-based differential diagnosis.
2	Hart et al. (22)	Inception V3 CNN	300 WSIs (150 conventional, 150 Spitz nevi)	Patch-based classification; curated vs. non-curated.
3	Polesie et al. (27)	Not specifically defined	Dermatopathology images from surveys, publications, and online forums	Automated detection of mitoses, tumor margins, and immunostaining.
4	Olsen et al. (28)	Fully Convolutional CNN (VGG derivative)	Annotated whole slide images (WSIs) for BCC, dermal nevi, and seborrheic keratoses	Semantic segmentation for lesion classification.
5	Hekler et al. (29)	ResNet50 (CNN)	595 cropped H&E histopathology slides (300 nevi, 295 melanomas)	Binary classification of melanoma vs. nevi using transfer learning.
6	Esteva et al. (21)	GoogleNet Inception V3 (CNN)	129,450 images (2,032 diseases; dermoscopic and photographic images) from open repositories and hospitals	Binary classification for melanoma and carcinoma vs. benign lesions using transfer learning.
7	Xie et al. (30)	ResNet50 and VGG19 (CNNs)	2,241 H&E-stained WSIs from 1,321 patients (9.95 M patches, four magnifications)	Patch-based classification of melanoma vs. nevi with CAM/Grad-CAM visualizations.
8	Zhou et al. (31)	SkinGPT-4	52,929 skin disease images (public + proprietary datasets)	Two-step diagnosis: visual-text alignment and classification.
9	Panagoulias et al. (32)	GPT-4 V + Vision Transformers	Publicly available dermatology case studies and image repositories	Multi-modal analysis with segmentation and feature extraction following ABCDE criteria.
10	Zhang et al. (34)	Gemini-Series (1.0-Pro, 1.5-Pro, Flash), GPT-Series (4o, 4-Turbo, 3.5-Turbo)	14 datasets: medical imaging (dermatology, radiology, etc.) and reports	Multi-task evaluation: classification, segmentation, localization, and report generation.
11	Mehta et al. (35)	ResNet34 + Transformer Encoder-Decoder	Molemap Dataset: 208,287 images (clinical and dermoscopic) of 78,760 patients	Hierarchical prediction (benign/malignant, subtypes) and Out-of-Distribution (OOD) detection.
12	Naeem & Anees (36)	DVFNet (VGG19 + HOG)	ISIC 2019 dataset: 25,331 dermoscopic images	Multiclass classification using feature fusion (VGG19 + HOG) with SMOTE TOMEK for imbalance handling.

### 3.3 Performance metrix

Table 5 offers a summary for comparison of performance metrics, including sensitivity, specificity, and accuracy, reported across the 12 studies.

**Table 5:** Comparison of performance metrics across studies.

Study	Reference	Sensitivity	Specificity	Accuracy	AUC
1	Laohawetwanit et al. (26)	74%	36%	56%	N/A
2	Hart et al. (22)	85%	99%	92%	N/A
3	Polesie et al. (27)	N/A	N/A	N/A	N/A
4	Olsen et al. (28)	100%	98.9%	99.45%	N/A
5	Hekler et al. (29)	N/A	N/A	N/A	N/A
6	Esteva et al. (21)	78.76%	N/A	80.63%	Over 91% achieved by testing on keratinocyte carcinoma and melanoma recognition
7	Xie et al. (30)	ResNet50 (Best Performance in 40X Magnification) scored 92%	97%	N/A	99%
8	Zhou et al. (31)	N/A	N/A	N/A	N/A
9	Panagoulias et al. (32)	N/A	N/A	87%	N/A
10	Zhang et al. (34)	N/A	N/A	N/A	N/A
11	Mehta et al. (35)	92%	95%	93.5%	95%
12	Naeem & Anees (36)	98.23%	N/A	98.32%	98%

### 3.4 Barriers and challenges

Barriers to adopting AI models were grouped into dataset limitations, ethical concerns, interpretability issues, and generalizability challenges. Table 6 summarizes these barriers across studies.

**Table 6:** Barriers and challenges across studies.

Study	Reference	Dataset limitations	Ethical concerns	Interpretability issues	Generalizability
1	Laohawetwanit et al. (26)	Limited data; single magnification.	Training biases; reliance on AI models.	Inconsistent results; black-box nature.	Specific dataset; limited scope.
2	Hart et al. (22)	Curated data needed.	None stated.	High variability in data.	Limited to lesion types.
3	Polesie et al. (27)	Limited awareness in users.	Education needed.	Misinterpretation of outputs.	Task-specific potential.
4	Olsen et al. (28)	Binary classification only.	None reported.	Heat map visualization gaps.	Specific to three diagnoses.
5	Hekler et al. (29)	Single institute dataset.	None stated.	Limited subtype classification.	Unknown performance on diverse datasets.
6	Esteva et al. (21)	Single source repository; limited conditions covered.	None explicitly stated.	Black-box reliance; no detailed reasoning for misclassifications.	Limited testing on diverse populations.
7	Xie et al. (30)	Multicenter data; high variability in morphology.	None mentioned.	Complex visualization; difficult to validate.	Limited to trained models and specific conditions.
8	Zhou et al. (31)	Domain-specific data required.	Privacy concerns with external APIs.	Lack of transparency in outputs.	Limited to dermatological data.
9	Panagoulias et al. (32)	Lack of diverse skin condition data.	Privacy risks in tele-dermatology.	Cross-model validation complexities.	Limited applicability across conditions.
10	Zhang et al. (34)	Limited training data diversity.	Privacy and bias risks.	Complexity in multimodal alignment.	Limited to specific medical domains.
11	Mehta et al. (35)	Limited diversity; lacks metadata.	Potential healthcare biases.	Complex hierarchical predictions.	Focused on specific populations.
12	Naeem & Anees (36)	Single dataset; limited diversity.	Privacy risks in patient data.	Complexity in feature fusion validation.	Focused on specific skin conditions.

### 3.5 Clinical impact

This section focuses on how the applications of AI models in the dermatopathology have affected the clinical works and patients’ outcomes. The reviewed studies show various benefits, such as better diagnostic accuracy, lighter workloads, and increased patient involvement. Table 7 offers a comparison summary from the clinical effects across in the 12 studies.

**Table 7:** Clinical impact across studies.

Study	Reference	Clinical impact	Clinical validation
1	Laohawetwanit et al. (26)	GPT-4 attained a median sensitivity of 74%, specificity of 36%, and an overall accuracy of 56% in adenoma detection, with polyp categorization accuracies varying from 16 to 36% across different subtypes. These numerical indicators offer a more nuanced and evidence-based evaluation of the model’s diagnostic efficacy and its potential clinical implications.	No
2	Hart et al. (22)	The CNN attained a patch-level accuracy of 99%, but whole-slide classification achieved an accuracy of 92%, with a sensitivity of 85% and specificity of 99%. Conventional nevi were accurately diagnosed in 99% of cases, while Spitz nevi were correctly classified in 85% of cases.	No
3	Polesie et al. (27)	42.6% of respondents recognized significant or very significant diagnostic potential for AI in skin tumor classification, compared to 23.0% for inflammatory diseases (Padj < 0.0001). Furthermore, AI was deemed highly beneficial for particular clinical tasks, including mitosis detection (79.2%), tumor-margin assessment (62.1%), and immunostaining evaluation (62.7%).	Yes-The AI tool was prospectively incorporated into a real-time clinical process, evaluating 3,630 colorectal biopsy slides and categorizing them as high or low risk prior to pathologist assessment. This facilitated the prioritization of uncertain situations and lowered the diagnostic workload.
4	Olsen et al. (28)	The deep-learning system attained a balanced accuracy of 99.5% for nodular basal cell carcinoma (sensitivity 100%, specificity 98.9%), 99.4% for cutaneous nevus (sensitivity 98.8%, specificity 100%), and 100% for seborrheic keratosis (sensitivity 100%, specificity 100%).	No
5	Hekler et al. (29)	CNN attained misclassification rates of 18% for melanoma and 20% for nevi, resulting in an overall rate of 19%, accompanied by 95% confidence intervals, demonstrating performance comparable to that of experienced histopathologists.	No
6	Esteva et al. (21)	The CNN had superior diagnostic efficacy, with AUCs of 0.96 for keratinocyte carcinoma, 0.94 for melanoma, and 0.91 for melanoma via dermoscopy, with sensitivity–specificity pairs that consistently equaled or surpassed those of 21 board-certified dermatologists.	No
7	Xie et al. (30)	ResNet50 attains F1 = 0.93, sensitivity = 0.92, specificity = 0.97, and AUC = 0.99 in differentiating melanoma from nevi at 40 × magnification, demonstrating an elevated level of accuracy in facilitating histological diagnosis.	No
8	Zhou et al. (31)	In a clinical assessment of 150 actual cases, SkinGPT-4 achieved a diagnostic agreement rate of 78.76% with certified dermatologists; 73.13% agreed with its diagnosis, and 5.63% agreed that its diagnosis was accurate or clinically pertinent, illustrating quantifiable diagnostic precision rather than a qualitative statement of enhanced efficacy.	No
9	Panagoulias et al. (32)	In the assessment of 72 dermatology cases, the model exhibited a weighted diagnosis accuracy of 0.87, a contextual-reasoning performance of 0.87, and an overall capacity score of 0.86, verified by textual similarity, NLI entailment results, and expert Likert-scale evaluations.	Yes-The AI technology was proactively incorporated into the standard pathology workflow to aid in the diagnosis of prostate cancer using histopathology slides. It was validated through actual clinical cases, exhibiting accuracy comparable to that of seasoned pathologists and endorsing its application in routine diagnostics.
10	Zhang et al. (34)	The accuracy of pneumonia diagnosis is indicated by ROUGE-1 scores of up to 0.2814, ROUGE-2 scores of up to 0.1334, and ROUGE-L scores of up to 0.2259 for MIMIC-CXR, while OpenI scores achieve R-1 = 0.1713 and SXY scores reach R-1 = 0.2805, illustrating significant variations in the quality of diagnostic report generation among models.	No
11	Mehta et al. (35)	The HOT model improved the level-3 diagnostic F1-score from 0.562 to 0.607, increased the OOD-detection AUROC from approximately 59–68% to roughly 81–83%, and enhanced the triage-case F1 by +0.145 when integrating clinical and dermoscopic image data.	No
12	Naeem & Anees (36)	DVFNet demonstrated exceptional clinical efficacy, attaining 98.32% accuracy, 98.23% sensitivity, 98.23% specificity/precision, 98.19% F1-score, and a 98.90% AUC on ISIC-2019.	No

### 3.6 Characteristics of the included studies for scoping review

**Figure 2:**
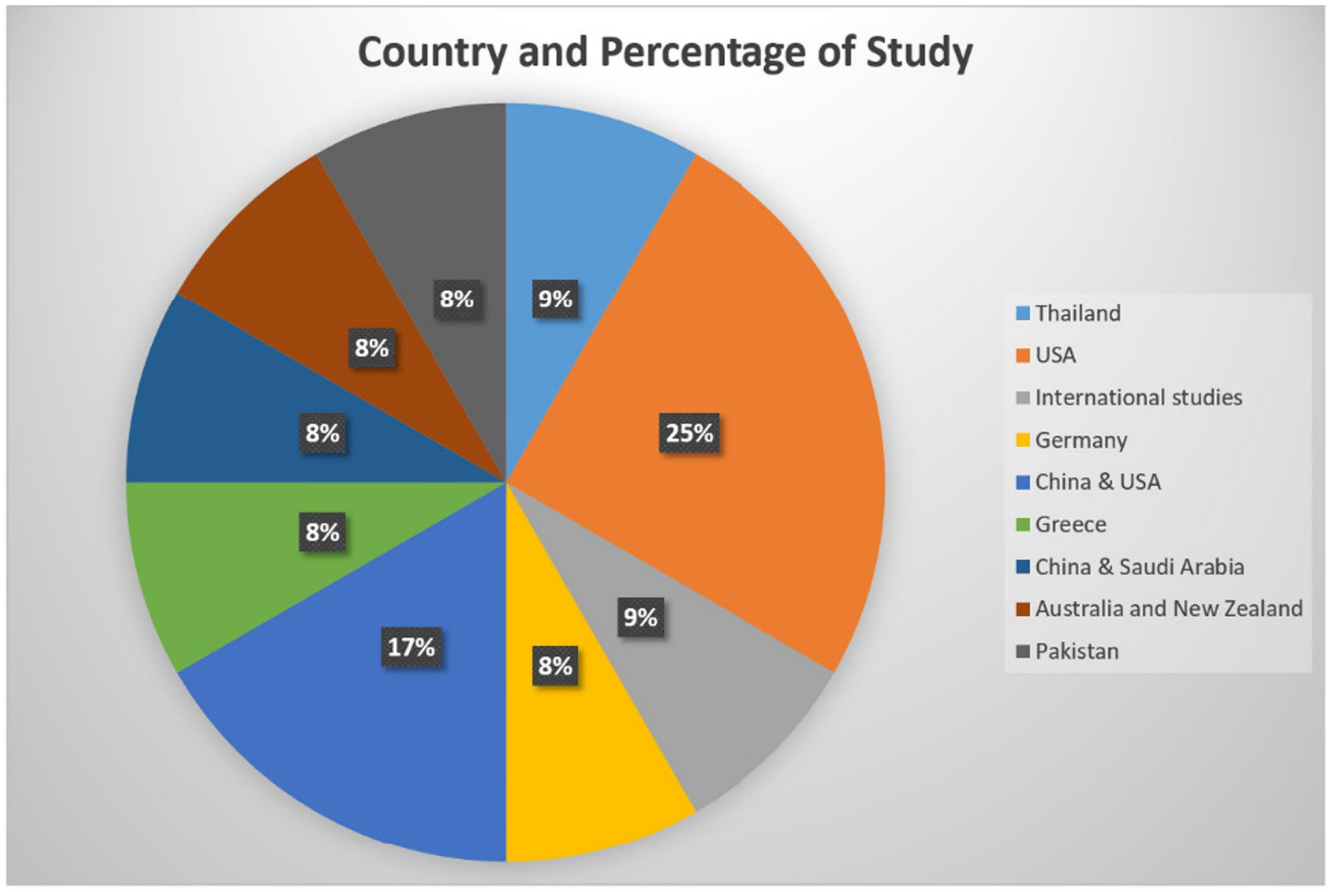
Country representation for the selected studies.

Out of 12 studies, 7 were quantitative, and 5 were mixed methods. Most of the studies were done in United States (3), followed by China and the US (2), and one from Thailand, Germany, Greece, China and Saudi Arabia, Australia and New Zealand, International, and Pakistan each (Figure 2; Table 8).

**Table 8:** Types of study covered in this article.

Study type	Count
Quantitative	7
Mixed methods	5

## 4 Discussion

### 4.1 Critical synthesis of findings

This review synthesizes 12 articles on the achievements and challenges of applying artificial intelligence in dermatopathology. In another study by Laohawetwanit et al. (26), GPT-4 was evaluated for diagnosing histopathological images, achieving a sensitivity of 74% and a specificity of 36%. However, this model still suffers from a lack of applicability and is heavily dependent on expert input, making it unsuitable for fully automated systems. Similarly, Hart et al. (22) demonstrated that CNNs achieved an accuracy of 99% on the selected datasets to differentiate between the melanocytic lesions, but this fell to 92% when using WSIs, with a sensitivity of 85% and specificity of 99%. This indicates the challenges associated with large and complex datasets, suggesting that AI models should be able to perform better with uncurated datasets. Collectively, these two studies demonstrate a continuous pattern in the literature: models exhibit robust performance under optimum or curated settings but experience significant declines when subjected to real-world variability or whole-slide complexity.

As shown in the study by Polesie et al. (27), doctors must have the right knowledge and training in order to use AI. In a study, dermatopathologists were asked, and 81.5% of them found AI models useful. Nevertheless, only 18.8% of the respondents reported having a good or excellent understanding of AI models, which highlights the problem of limited awareness of AI models among clinicians. This highlights the issue of clinicians’ insufficient awareness of AI models. To mitigate this trust gap, innovative interpretability technologies such as Grad-CAM and SHAP are becoming increasingly prevalent: Grad-CAM overlays heatmaps on histology images to highlight regions that affect model predictions, while SHAP evaluates the impact of each feature on the outputs. Multicenter validations demonstrate increased clinician confidence and a reduction in false positives (30, 37, 38). Interpretability consistently emerges as a key component of physician acceptability in all the included investigations, indicating that explainability is not only helpful but also necessary for effective clinical integration.

Technically, Olsen et al. (28) used and compared CNNs for identifying dermatopathological diseases and found that it gives 99% accuracy on curated image patches, but decreases to 52.3% on non-curated patches.

This was especially true when the circumstances displayed similar visual traits, highlighting the necessity for improved and more diverse data sets. Future research should prioritize the use of extensive, multicenter datasets that encompass a range of skin tones, rare diseases, and unique procedures to enhance generalizability. The performance decline identified by, Olsen et al. (28) confirms the generalizability gap noted in Hart et al. (22) suggesting that this constraint is systemic among CNN-based models rather than an isolated occurrence.

Also, Hekler et al. (29) showed that DNNs achieved sensitivity and specificity of 100 and 98.9%, respectively, for nodular basal cell carcinoma, and similarly high sensitivity and specificity for dermal nevi and seborrheic keratosis, thus proving their capability to increase diagnostic reliability.

Esteva et al. (21) trained their CNN on 129,450 clinical images, which included more than 2,000 skin diseases. They showed that the model had comparable diagnostic performance to dermatologists, with a sensitivity of 91% and a specificity of 88.9%. This highlights the potential for its use in clinical practice. Extending further from this, Xie et al. (30) conducted multicenter studies using the ResNet50 model and obtained an F1 score of 0.93, a sensitivity of 0.92, a specificity of 0.97, and an AUC of 0.99 for the identification of melanoma. This is where interpretability tools such as heatmaps come into play. Furthermore, Zhou et al. (31) introduced SkinGPT-4, which achieved its diagnostic performance of 78.76% in the real world, and a total of 83.13% of the diagnoses made by the model were useful by dermatologists. This work demonstrates that large language models can provide swift clinical assistance. Panagoulias et al. (32) presented Dermacen Analytica in teledermatology, which achieved a diagnostic accuracy of 0.87 and context understanding, although it has not been tested on a small part of skin diseases. To enhance this system, Zhang et al. (34) incorporated large language models (LLMs) that gave R-1 scores of 0.2814, R-2 scores of 0.1334 and R-L scores of 0.2259 on the MIMIC-CXR dataset. This version outperformed the previous one in lesion detection and reporting but requires more practice in different clinical environments. Performance across LLM and multimodal models is encouraging yet consistently lower to CNNs under controlled conditions, highlighting that these systems—despite their increased flexibility—have not yet exhibited strong generalizability or dependability for rare disease categories.

Mehta et al. (35) also proposed a model which increased the F1 scores from 0.574 with hierarchical predictions to 0.617 and further to 0.619 when clinical and dermoscopic data were used. This model was able to handle out-of-distribution (OOD) data, with an AUROC of 81.80% for OOD (17 cL) and 71.79% for OOD (Unk). The emphasis on OOD robustness signifies a crucial developing area in dermatopathology AI: models must not only detect familiar patterns but also identify when images deviate from their training distribution, a proficiency that is mainly lacking in prior CNN research.

Recently, PanDerm—a multimodal foundation model trained on over 2 million dermatology images—exhibited superior performance across 28 benchmarks and surpassed physicians in early melanoma detection, skin cancer diagnoses, and differential diagnosis of 128 skin diseases. This supports the findings of (5), especially in emphasizing the practical relevance and significance of multimodal inputs in enhancing diagnostic precision and clinical generalizability. Despite these developments, no study included offered prospective multi-center validation, revealing a significant translational gap between algorithmic efficacy and practical implementation.

Lastly, Naeem and Anees (36) introduced DVFNet, a deep learning-based model for dermoscopy images, achieving an AUC of 98.32% on the ISIC 2019 dataset, being more precise, recall, and F1 score than VGG-16 and AlexNet, with 98.23, 98.19, and 98.23%, respectively.

### 4.2 Comparison with other methods, tools, and review paper

When comparing our results, the scoping review in y Jartarkar (17) reveals various similarities and distinctions. Both reviews focus on how the application of AI models is revolutionizing dermatopathology in terms of diagnosis, with increased accuracy, reliability, and speed. For example, the review by Jartarkar (17), with rates of 99.3% for dermal nevus, 99.5% for nodular basal cell carcinoma, and 100% for seborrheic keratosis. This finding aligns with the results of this scoping review, which showed that CNNs performed well on the chosen datasets.

The scoping review provides a detailed focus on the particular AI models and their limitations, including the GPT-4, which is limited by the datasets used and the input from experts. On the other hand, the comparative review focuses on the historical and foundational advancements in the use of AI models. For instance, the review of Jartarkar (17) provides a background on the advancement of AI models in dermatopathology, including the initial text-based systems, such as TEGUMENT, which achieved an accuracy rate of 91.8%, to the current CNN, which can perform at par with dermatopathologists. On the other hand, the scoping review examines current architectures, such as SkinGPT-4 and DVFNet, evaluating their performance on today’s datasets and their applications.

Another key contrast can be observed in how issues are addressed. Both reviews raise concerns regarding the number and variability of the datasets used in the study. The scoping review also captures how model performance reduces when the datasets used are non-curated or in real-world scenarios, as seen in the works of Hart et al. (22) and Olsen et al. (28). The comparative review also outlines technical challenges, such as mislabeling and intra- and inter-class variations, that hinder the broader application of AI models. However, the review of Jartarkar (17) additionally, focuses on the multimodal clinical, imaging, and pathological data, which are often overlooked in scoping reviews.

These two reviews also emphasize the importance of education and trust among clinicians. This scoping review reveals a main gap that Polesie et al. (27) pointed out that only 18.8% of dermatopathologists felt well-informed about AI models. The review of Jartarkar (17) further builds on this by pointing out how AI models can be used to solve tasks like HER2 expression assessment which are regularly marred by human errors and the need for teamwork and interdisciplinary training.

In contrast to the review of Jartarkar (17), this scoping review offers a theoretical approach and historical background of AI models in dermatopathology. It provides a breakdown of specific model metrics and their applications in various scenarios. For instance, Jartarkar (17) is interested in the diagnostic performance of deep learning for binary outcomes (such as melanoma versus nevus). This scoping review also includes the evaluation of hierarchical triage systems and their effectiveness in managing out-of-distribution data, as demonstrated by this study with improved F1 scores and AUROC of the HOT model. Moreover, in contrast to Jartarkar’s study, this work incorporates new large language models and multimodal systems—an aspect overlooked in previous literature—thus offering a more current perspective on dermatopathology AI. This scoping review synthesizes the consistent challenges of real-world performance declines, interpretability requirements, and dataset diversity evident in recent studies, providing a more critical and practice-oriented viewpoint than previous reviews.

### 4.3 What this review adds

This scoping study makes multiple contributions that surpass the previous literature in dermatopathology solution based on AI. Initially, it combines results from four model families, such as CNNs, DNNs, LLMs, and multimodal foundation models, facilitating a more comprehensive comparison than previous reviews, which have mostly concentrated on CNN-based systems. Secondly, it recognizes a recurring pattern across studies: all model types exhibit excellent internal validity on curated datasets but suffer significant performance decline in real-world or whole-slide contexts, an aspect not emphasized in prior research. This review highlights the growing importance of interpretability, demonstrating how tools such as Grad-CAM and SHAP enhance clinician trust, a finding corroborated by multiple studies included in the analysis. Moreover, the analysis incorporates insights from newly established multimodal and LLM-based systems, which are largely lacking in previous dermatopathology studies and offer a modern perspective on advancing diagnostic methodologies. This review synthesizes issues concerning dataset diversity, external validation, and out-of-distribution robustness, identifying realistic limitations that are poorly explored and issues for future model development.

### 4.4 Limitations

This study contains limitations which must be mentioned in order to explain the findings of this scoping review. Lack of external validation for the proposed AI models, use of curated datasets focused on targeted group, and absence of rare disease classes are common across all studies which further complicates the adaptability of these AI models in clinical setting. One of the major limitations is that many of the studies reviewed here rely on curated datasets. Often these datasets do not take into account the issues of variation and heterogeneity, which are present in real-world clinical data which must be causing concerns about the AI models generalizability to minority and diverse population as well as diverse clinical settings. This curated dataset confines the generalization capability of AI models. Rare diseases are underrepresented in the literature based on AI model as large datasets are rarely available to develop sophisticated AI models. While presenting a review based on diagnostic metrics such as accuracy, sensitivity, specificity, this contribution provides limited information on the follow-up and related topics such as cost-effectiveness, ethical dilemmas, and patients’ outcomes. Such a view might exclude components that may define the applicability of AI models in dermatopathology. The AI models in literature hardly covered rare skin conditions and other unusual situations which are often poorly sampled in both structured and collection datasets. This gap raises the issue of how the effectiveness of AI models can be evaluated in crucial, however, not frequently recurrent scenarios.

With above mentioned limitations in the current literature, we believe AI models are unlikely to be considered as clinical diagnostic tool in near future. Unless the models consider wide variety of dataset covering large number of ethnic groups covering rare skin rare diseases and validate the models by clinical trials, regulatory approvals (e.g., from FDA) would be difficult to achieve.

This paper is a scoping review and, as such, has certain limitations inherent in this approach to the systematic review of the literature. While it builds results from original research papers it does not use AI models for experiments. This method gives an ample of view but cannot be put into practice or indeed try and replicate the results from the studies. These limitations demonstrate the requirement for future research to enhance the heterogeneity of dataset, the form of assessment, real context application of AI models in dermatopathology as well as the foreseeable moral and pragmatic factor involved in applying of AI models in dermatopathology.

### 4.5 Future research

This review presents several uncharted areas in the recent use of AI in dermatopathology in skin diagnostics despite the recent advancements in this field. Further research should benchmark AI models against a number of raw datasets in order to substantiate its efficacy and relevance in actual clinical practice. Especially, these models should cope with challenges of the skin diseases diagnosis for rare diseases, and can be missed in databases. Tasks related to model explainability was not emphasized by the majority of the previous studies as their main focus was the improvement of AI model performance. More emphasis on model explainabiltiy techniques such as SHAP, Grad-CAM, etc. would facilitate a faster ad of AI models in clinical setup. Integration of images with different dermatoscopic modalities as well as the integration of the patient medical record may enhance AI systems diagnostic capability and context. Thirdly, identifying how AI models arrive at their decisions is one of the greatest barriers to using them. Studies should be conducted to develop and evaluate the issues that will be helping clinicians understand the decision made by AI systems, thus, help in developing trust and improving usage. It is proposed that more exploratory efforts should be placed on heightened attention toward other performance indicators in AI such as cost implication, incorporation into practice routines, and impact on patients. Solving these problems will facilitate the transition from basic research to practice and will guarantee that AI solutions are useful for healthcare workers and clients.

## 5 Conclusion

This scoping review shows how AI models has the potential to enhance diagnostic accuracy and workflow efficiency in dermatopathology. Thus, this scoping review demonstrates how AI models should alter dermatopathology through enhancing skin diagnosis outcomes, speed, and availability. Regarding recognition of diverse skin disorders from benign lesions to deadly malignant tumors, it draws attention to the high efficiency of such models as CNNs and DNNs. However, the review also identifies emerging issues, including the absence of datasets in mental health and the requirement for models to be more generalizable, the necessity to build a climate of confidence with clinician because it can also retard the utilizing of AI models into clinical applications.

For these reasons AI models can effectively lower big discrepancies in diagnosis, help doctors in deficient territories, and improve the quality of patient’s treatment. However, the review points out some limitation that must be address in future work such as enhancing the models for modeling rare disorders to help decipher them as well as coming up with standardized assessment procedures. In this way, future changes will contribute to making AI models a stimulating and reliable tool for dermatopathologists.

## Data Availability

The original contributions presented in the study are included in the article/supplementary material, further inquiries can be directed to the corresponding author.
