# Correction: Revolutionizing dermatopathology using AI in skin diagnostics: scoping review

**DOI:** 10.3389/fmed.2026.1816491

**Published:** 2026-02-26

**Authors:** Frontiers Production Office

**Affiliations:** Frontiers Media SA, Lausanne, Switzerland

**Keywords:** AI, CNN, dermatology, LLM, ViT

An incorrect **Funding** statement was provided. The correct funding statement reads:

“The author(s) declared that financial support was received for this work and/or its publication. Open access publication of this article was supported by the College of Science and Engineering, Hamad Bin Khalifa University (HBKU), Qatar.”

The original version of this article has been updated.

